# Comparative Proteomic Study of Fatty Acid-treated Myoblasts Reveals Role of Cox-2 in Palmitate-induced Insulin Resistance

**DOI:** 10.1038/srep21454

**Published:** 2016-02-22

**Authors:** Xiulan Chen, Shimeng Xu, Shasha Wei, Yaqin Deng, Yiran Li, Fuquan Yang, Pingsheng Liu

**Affiliations:** 1 Laboratory of Protein and Peptide Pharmaceuticals & Laboratory of Proteomics, Institute of Biophysics, Chinese Academy of Sciences, Beijing 100101, China; 2 National Laboratory of Biomacromolecules, Institute of Biophysics, Chinese Academy of Sciences, Beijing, 100101, China; 3 University of Chinese Academy of Sciences, Beijing, 100101, China; 4 Department of Biological Science and Biotechnology, School of Biological Science and Medical Engineering, Beihang University, Beijing, 100191, China

## Abstract

Accumulated studies demonstrate that saturated fatty acids (FAs) such as palmitic acid (PA) inhibit insulin signaling in skeletal muscle cells and monounsaturated fatty acids such as oleic acid (OA) reverse the effect of PA on insulin signaling. The detailed molecular mechanism of these opposite effects remains elusive. Here we provide a comparative proteomic study of skeletal myoblast cell line C2C12 that were untreated or treated with PA, and PA plus OA. A total of 3437 proteins were quantified using SILAC in this study and 29 proteins fall into the pattern that OA reverses PA effect. Expression of some these proteins were verified using qRT-PCR and Western blot. The most significant change was cyclooxygenase-2 (Cox-2). In addition to whole cell comparative proteomic study, we also compared lipid droplet (LD)-associated proteins and identified that Cox-2 was one of three major altered proteins under the FA treatment. This finding was then confirmed using immunofluorescence. Finally, Cox-2 selective inhibitor, celecoxib protected cells from PA-reduced insulin signaling Akt phosphorylation. Together, these results not only provide a dataset of protein expression change in FA treatment but also suggest that Cox-2 and lipid droplets (LDs) are potential players in PA- and OA-mediated cellular processes.

Like glucose, blood free fatty acids (FFAs) can serve as a readily available energy source, but similar to glucose, at a high concentration can lead to serious detrimental health consequences. High circulating FFA, especially saturated forms, plays a key role in inducing metabolic syndromes such as insulin resistance. Skeletal muscle, which utilizes about 75% of the total body glucose budget, is also highly responsive to insulin signaling and a major consumer of FFAs as fuel^1^,^2^. Previous studies have shown that saturated FFAs inhibit insulin sensitivity of skeletal muscle cells while unsaturated FFAs counteract this inhibitory effect^3^,^4^,^5^,^6^.

Mechanistic studies have revealed some putative pathways linking FFAs to insulin signaling. For example, the saturated fatty acid palmitic acid (PA) induces endoplasmic reticulum (ER) stress and insulin resistance, while treatment with the unsaturated fatty acid oleic acid (OA) or with a chemical chaperone relieves stress and restores insulin sensitivity. Palmitate-mediated production of the protein kinase C activator, diacylglycerol (DAG), and another second messenger, ceramide, is also involved in the suppression of insulin signaling^7^,^8^. A possible downstream element liking these two mediators is the inhibition of insulin receptor substrate 1 (IRS-1). Lastly, recent studies suggested that DAG plays distinct roles in the regulation of insulin signaling, depending on its cellular location^9^,^10^.

Ectopic lipid accumulation has emerged as being strongly associated with metabolic syndromes, in particular in insulin resistance^11^. Lipid droplets (LDs) are the cellular organelle responsible for the storage of neutral lipids such as triacylglycerol (TAG), cholesterol ester (CE), ether lipids, and other fatty acid esters^12^. Excessive accumulation of neutral lipids in this organelle has been implicated in many metabolic diseases, including obesity, atherosclerosis, liver steatosis, and type 2 diabetes mellitus (T2DM)^13^,^14^,^15^. In particular, the accumulation of intramuscular TAG (IMTG) in LDs has been well correlated with the development of insulin resistance in skeletal muscle^16^,^17^.

LDs are central to the regulation of FFA levels within the cell balancing supply, from synthesis to absorption, and demand from anabolism to respiration. With the exception of their use in membrane lipid synthesis, FFAs absorbed by cells are incorporated into TAG or other FA esters and stored in LDs, reducing cellular toxicity from excess FFAs. Thus, through this mechanism, LDs can reduce lipid-mediated toxicity, however, the accumulation of LD lipids is also associated with insulin resistance. When energy is required, FFAs are then released from LDs through the action of lipases such as adipose TAG lipase (ATGL) and hormone sensitive lipase (HSL), and are utilized in mitochondria for β-oxidation. The analysis of the impact of LDs on insulin signaling may provide insights into the molecular mechanisms linking ectopic lipid storage to metabolic disorders.

Some metabolism-related genes are regulated by saturated FFAs in skeletal muscle cells, providing clues linking FFAs to insulin resistance. Among them is cyclooxygenase-2 (Cox-2) which is upregulated by PA^18^. Furthermore, The Cox-2 activity has been reported to influence insulin sensitivity^19^,^20^. In leukocytes, Cox-2 is localized on LDs and plays important roles in inflammation^21^. Inducible Cox-2 has been also found on LDs in other cell types such as colon cancer cells^22^. Therefore, it is possible that Cox-2 may be part of mechanism linking FFAs, LDs and insulin signaling. In order to identify proteins that are regulated by FFAs, we conducted a comparative proteomic study using C2C12 myoblasts cultured in the presence of PA or PA plus OA. We found that Cox-2 was the protein most responsive to these treatments and, importantly, a Cox-2 inhibitor could block PA-induced insulin resistance.

## Results

### Quantitative proteomic study of fatty acid-treated myoblasts

Previous studies have demonstrated that saturated fatty acid PA could induce insulin resistance in skeletal muscle^3^,^4^, while unsaturated fatty acid OA is able to reverse the inhibitory effect of PA on the insulin signaling pathway^4^. However, the protein(s) that play important roles in OA-induced insulin sensitization remain to be identified. To dissect potential molecular mechanisms behind the effects of OA, we employed an unbiased SILAC-based quantitative proteomic approach to identify changes of protein expression in C2C12 myoblasts following treatment with PA and PA plus OA.

**Figure 1:**
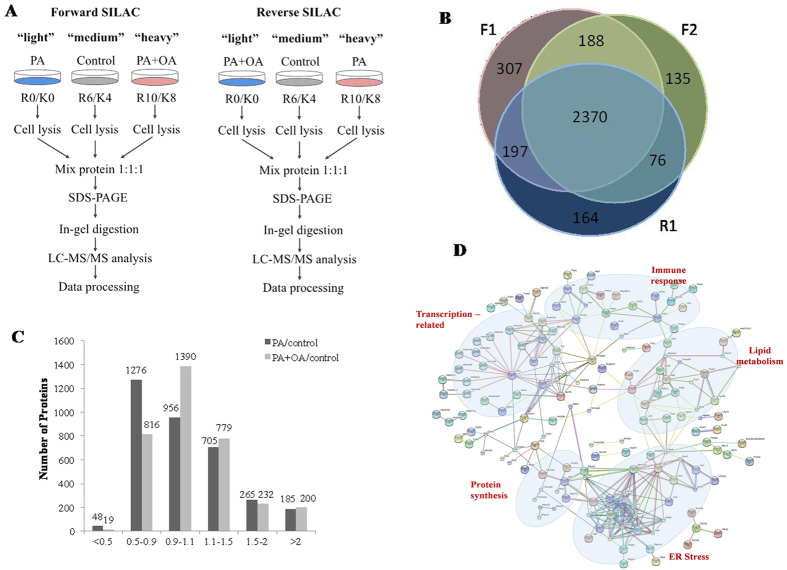
SILAC-based comparative proteomic analysis of fatty acid-treated myoblasts. (**A**) Flowcharts of forward SILAC and reverse SILAC labeling combined with LC-MS/MS for the comparative analysis of protein expression in C2C12 myoblasts upon PA and PA + OA treatment(R0/K0: normal isotopes, K4/R6: [^2^H_4_]lysine/[^13^C_6_] arginine, K8/R10: [^13^C_6_,^15^N_2_]lysine/[^13^C_6_,^15^N_4_] arginine). (**B)** Quantitation overlap of the quantified proteins in the two forward and one reverse SILAC labeling experiments (F1&F2: forward experiment, R1: reverse experiment). (**C**) The distribution of expression ratios for the quantified proteins, including those quantified in only one set of SILAC labeling experiment, and the mean protein expression ratios were used for the data quantified in at least two sets). (**D**) (STRING database mapping the interaction of the differentially expressed proteins upon the treatment of PA and PA + OA. These proteins form several apparent interaction groups exerting diverse biological functions including ER stress, lipid metabolism, immune response, gene transcription and protein synthesis.

**Figure 2:**
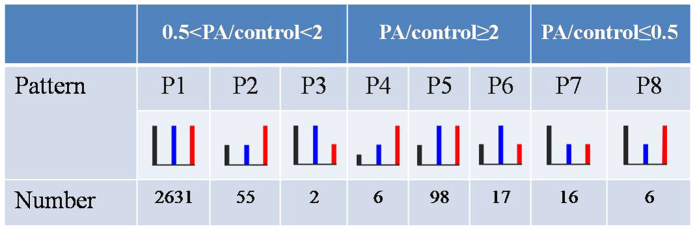
Eight protein expression patterns classified according to their different responses to PA and PA + OA treatments. Proteins were first grouped into three major categories based on their response to PA treatment: up-regulated (PA/control ≥ 2), down-regulated (PA/control ≤ 0.5), and unchanged (0.5 < PA/control < 2). Within each category, the proteins were further sorted into different subcategories according to their responses to PA + OA treatment.

To obtain reliable quantitative results, SILAC experiments were conducted in triplicate, including two forward and one reverse labeling (Fig. 1A depicts the procedure for SILAC-based quantitative proteomics).Using LC-MS/MS analysis we quantified 3062, 2769 and 2807 proteins in the two forward and one reverse SILAC experiments, respectively. Nearly 70% of the proteins were quantified in all the three replicates, and 82.4% proteins were quantified at least twice in the three replicates (Fig. 1B), implying a good level of reproducibility of the MS analysis. A total of 3437 distinct proteins were quantified in this study (Supplemental Table S1). An overview of the changes in protein expression levels from PA and PA plus OA treatment is presented in Fig. 1C. The expression level of the majority of proteins was unchanged upon PA or PA plus OA treatment. The mean experimental to control ratios of the proteins quantified more than twice were 1.11 ± 0.26 and 1.14 ± 0.17 for PA/Control and PA + OA/Control, respectively (mean ± SD). Therefore, a two-fold change was chosen as the threshold for a significant change in protein expression. To improve the reliability of the results, only the proteins quantified in at least two of the three SILAC labeling experiments were considered for further functional analysis. Based on these criteria, eight patterns were defined and the results in relation to these patterns are lists in Supplemental Table S2 and summarized in Fig. 2.

Proteins were first grouped into three major categories based on their response to PA treatment: up-regulated (minimal ratio (PA/control) of 2), down-regulated (maximal ratio (PA/control) of 0.5), and unchanged (0.5< ratio(PA/control) <2). Within each category, the proteins were further sorted into different subcategories according to their responses to PA plus OA treatment. Pattern 1 (P1), into which the majority of proteins (92.9%) fell, was not influenced by PA or PA plus OA treatment. Pattern2 (P2), pattern 5 (P5), pattern 6 (P6), and pattern 7 (P7) were the major response patterns for the treatment with PA or PA plus OA in C2C12 myoblasts. In P2, the expression of proteins was only responsive to the treatment of PA plus OA. The proteins in P5 were up-regulated following the treatments of PA and PA + OA. That is, OA did not reverse the up-regulation effect induced by PA. As previous studies have shown that OA can reverse the insulin signaling inhibition induced by PA, we focused our attention on patterns P4, P6, and P8 in which OA treatment resulted in an enhancement or reversal of the effect of PA treatment. A representative protein list is shown in Table 1.

To better understand the overall influence of fatty acids on the signaling network, we looked for known protein interactions among all the proteins that were differentially expressed upon treatment with PA or PA plus OA. We used the Search Tool for the Retrieval of Interacting Genes/Proteins (STRING) database, which is constructed on the basis of both physical and functionalinteractions^23^,^24^. Several interaction groups are immediately apparent (Fig. 1D). One core group of proteins, such as Sec11a, Ssr1, Ssr3, Sec61a1, and Spcs2, are involved in protein folding and transport in the ER. The regulation of these ER stress-related proteins by fatty acid treatment agrees with previous finding that the ER stress pathway is a potential mechanism for lipid-induced insulin resistance^25^. The regulation of a group of lipid metabolism-related proteins, including fatty acid desaturases (Fads1/2), fatty acid elongase (Elovl1), and acyl-CoA thioesterases (Acot1/2), make sense with the application of fatty acids. Other interaction subgroups suggest potential connections between fatty acid treatment and diverse biological functions, such as immune response, gene transcription, and protein synthesis.

### Verification of the SILAC based quantitative proteomic results

**Figure 3:**
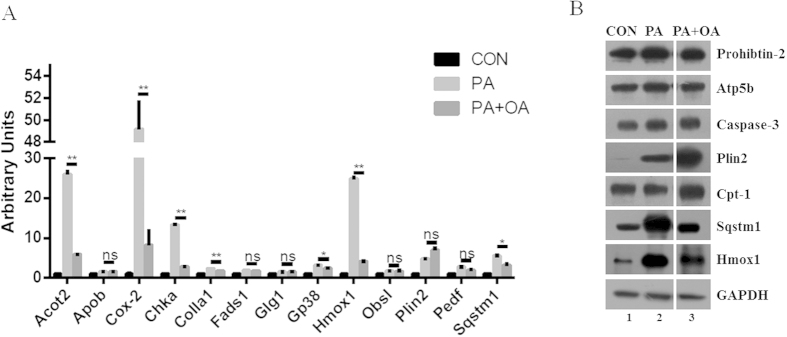
Palmitate and oleate mediate protein expression in myoblasts. 95% confluent C2C12 cells were treated with ethanol vehicle, 500 μM PA, or 500 μM PA plus 200 μM OA in DMEM with 10% FBS for 12 h. (**A**) Several genes were chosen from Patterns 4, 6 and 8 (see Table 1) to assess the accuracy of the SILAC findings. Expression of Acot2, Cox-2, Chka, Gp38, Hmox1, Plin2, Pedf, and Sqstm1 were determined using RT-PCR. Data were presented as mean ± SEM (n = 3) and compared by one-way ANOVA. *0.01 < P < 0.05. **P < 0.01. (**B**) Several proteins from Patterns 1, 4, 5, and 6 were chosen for Western blot analysis to verify the SILAC and RT-PCR results, including Prohibitin, Atp5b, Caspase-3, Plin2, Cpt-1, Sqstm1, and Hmox1. CON stands for control.

To confirm the accuracy of SILAC based quantification, several genes belonging to pattern P4, P6, and P8 were selected for validation using qRT-PCR (Fig. 3A). In agreement with the SILAC results, the expression of Plin2 was enhanced in both fatty acid treatment regimens. The expression patterns of genes for several other proteins confirmed the SILAC results, including Acot2, Cox-2, Chka, Colla1, Gp38, Hmox1, and Sqstm1 (Table 1 and Fig. 3A). In addition, several proteins belonging to patterns P1, P4, P5, and P6 were also selected for confirmation using Western blotting (Fig. 3B). These qPCR and Western blotting results were in conformity with the ratios obtained with SILAC, validating the accuracy of our SILAC results.

### Isolation of lipid droplets from fatty acid-treated C2C12 cells

IMTG has been correlated with insulin resistance in skeletal muscle^26^. Fatty acids with different saturation levels have different effects on the accumulation of lipid in muscle cells. PA induces much more phospholipid and DAG formation, while OA causes much more accumulation of TAG stored in LDs^3^,^4^. This differential response suggests that LDs may be involved in insulin sensitivity. Therefore, we examined the changes in protein concentrations in response to fatty acid treatments, specifically on LDs. Combining these comparative proteomic analyses may provide useful clues to find out how FFA saturation levels affect on the accumulation of IMTG and the relationship between LDs formation and insulin resistance.

**Figure 4:**
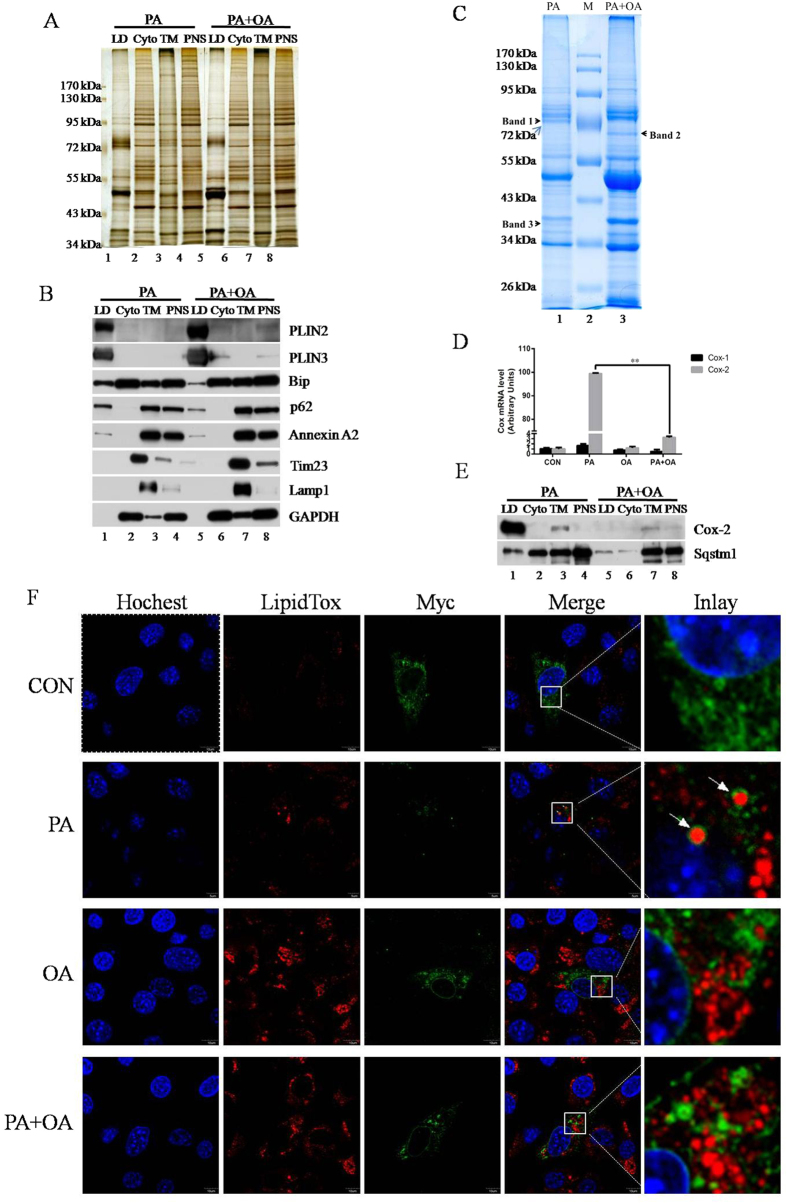
PA stimulates Cox-2 expression on LDs. For LD purification, 10^9^ C2C12 cells were treated with 500 μM PA or 500 μM PA plus 200 μM OA in DMEM with 10% FBS for 12 h. (**A)** Silver staining to assess the purity of LDs. The profile of LDs was dramatically different from the other three fractions. (**B)** Different cellular component marker proteins were probed byWestern blot. The LDs had little signal from other organelle marker proteins except ER, which suggests that LDs were physically associated with ER membrane. (**C**) LDs from different FFA-treated C2C12 cells were analyzed by Colloidal blue staining after SDS-PAGE separation. Three major bands showing differential expression were isolated for mass spectrometry analysis. (**D**) 95% confluent C2C12 cells were treated with ethanol vehicle, 500 μM PA, 200 μM OA,or 500 μM PA plus 200 μM OA in DMEM with 2% BSA for 12 h. Data were presented as mean ± SEM (n = 3) and compared by one-way ANOVA.**P < 0.01.(**E**) The expression of Cox-2 and Sqstm1 in all cellular fractions in different FFA treatments was determined using Western blot. (**F**) Transfected C2C12 cells were cultured on coverslips in 24-well plates. The cells were treated with ethanol vehicle, 500 μM PA, 200 μM OA, or 500 μM PA plus 200 μM OA in DMEM with 10% FBS for 12 h after24 h post-transfection and then subjected to immunofluorescence. Bar = 10 μM.

LDs were isolated from C2C12 myoblasts that were treated with different FFAs by a modified method, described previously^27^.The purity of the isolated LDs was examined by comparing proteins between LDs and other cellular fractions using silver staining after SDS-PAGE separation as well as by Western blot with cytosolic and other cellular faction marker proteins (Fig. 4A,B).As shown in Fig. 4A, proteins extracted from isolated LDs displayed a relatively simple pattern that was obviously distinct from the protein profiles of the other three cellular fractions, including post-nuclear supernatant (PNS), total membranes (TM), and cytosol (Cyto). Besides the unique protein pattern, the purity of isolated LDs was further confirmed by the relative enrichment of LD-resident proteins adipose differentiation related protein (ADRP, PLIN2) and tail interacting protein of 47 kDa (Tip47, PLIN3), and the near absence of markers that correspond to other intracellular compartments, such as lysosome protein Lamp1, cytosol protein GAPDH, mitochondrial membrane protein Tim23, and plasma membrane protein Annexin A2 (Fig. 4B). Small amounts of two ER proteins, p62 (ER membrane protein) and GRP78 (ER luminal protein) were found in the LD fraction^28^,^29^. Interestingly, OA treatment reduced both LD-associated ER proteins, suggesting good purity of the LDs. Collectively, these results suggest that the isolated LD fraction from C2C12 cells was largely free of contamination except for ER.

The LDs from cells treated with both fatty acids (PA + OA) had more PLIN 2 and PLIN 3 than cells treated only with PA (Fig. 4B, lanes 1 and 5), which is consistent with the result shown in Fig. 3A,B. A reduction of both ER proteins Bip and p62 in the PA + OA treated LDs (Fig. 4B, lanes 1 and 5) is suggestive of reduced inter-organelle contact between LDs and ER under these treatment conditions, which is in agreement with our previous report^4^.The LDs isolated from cells from either condition were free from contamination by the lysosomal and cytosolic proteins Lamp1 and GAPDH (Fig. 4B). In contrast, it is possible that an interaction between mitochondria and LDs was induced by OA treatment, as suggested by the presence of a weak band of Tim23 in LDs from PA + OA treated cells (Fig. 4B, lane 5).

### Alteration of lipid droplet proteins after fatty acid treatment

To further study the dynamics of LD proteins following FFA treatment, the LD proteins from FFA-treated cells were separated by SDS-PAGE and compared using Colloidal blue staining (Fig. 4C). Consistent with the silver staining and Western blot results (Fig. 4A,B), the intensity of the band containing PLIN2 and PLIN3 was dramatically enhanced in PA + OA-LDs (Fig. 4C). Among many other visible alterations between PA- and PA + OA-LDs, three protein bands were significantly distinct. Two unique protein bands appeared following PA treatment while a third prominent band was present following PA + OA treatment (Fig. 4C, arrow). These three bands were then sliced and subjected to mass spectrometry analysis. The results are shown in Supplemental Table S3 and proteins identified with more than 5 peptides were listed in Table 2.

Among the diverse proteins detected, prostaglandin G/H synthesis 2 (Cox-2) was found as the most abundant protein in band 1. Cox-1 was also identified in band 1. A qPCR experiment was conducted to determine whether Cox-1 expression was up-regulated in PA-treated cells since it was not detected in our SILAC analysis (Table 1). As shown in Fig. 4D the expression of Cox-2, but not Cox-1, was dramatically altered by the different fatty acid treatments. Western blotting further demonstrates that PA-LDs had a much higher expression ofCox-2 than PA + OA-LDs (Fig. 4E, lanes 1 and 5). Collectively, these results demonstrate that Cox-2 is dramatically upregulated by PA treatment and this treatment results in increase expression of Cox-2 specifically at the LDs in C2C12 cells. PA-induced Sqstm1 was reduced by OA and was primarily located in the TM faction, which was used as a control (Fig. 4E). To verify this finding, Myc-tagged Cox-2 was transiently expressed in C2C12 cells and the cells were treated with different FFAs. After the treatments, immunofluorescence with anti-Myc antibody was conducted. Using LipidToxRed and FITC-conjugated anti-mouse IgG to stain LDs and Cox-2 respectively, Myc-Cox-2 was found on LDs in PA-treated C2C12 cells (Fig. 4F).

### Inhibition of Cox-2 recovers PA-reduced Akt phosphorylation

**Figure 5:**
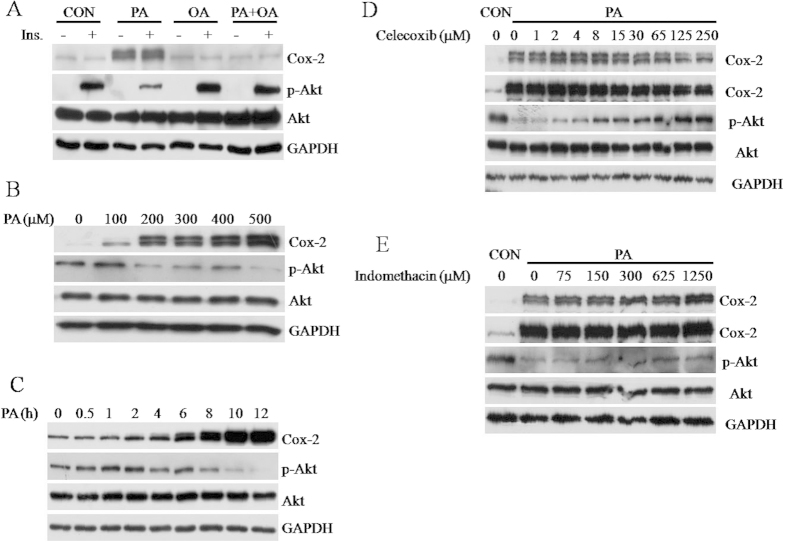
Cox-2 mediates palmitate-induced suppression of Akt phosphorylation. (**A)** 95% confluent C2C12 cells were treated with ethanol vehicle, 500 μM PA, 200 μM OA, or 500 μM PA plus 200 μM OA in DMEM with 2% BSA for 12 h. Cox-2 and p-Akt were examined using Western blot. GAPDH was used as an internal protein loading control. (**B,C**) 95% confluent C2C12 cells were incubated in the presence of different concentration of PA (0,100 μM, 200 μM, 300 μM, 400 μM, 500 μM) in DMEM with 2% BSA for 12 h or in the presence of 500 μM PA in DMEM with 2% BSA for the indicated time. (**D,E)** Inhibition of Cox-2 reduced PA-induced insulin resistance. 95% confluent C2C12cells were treated with ethanol vehicle (CON) or 500 μM PA in DMEM with 2% BSA for 12 h. The concentrations of inhibitors used are indicated; otherwise the highest concentration was used.

Both the SILAC and the comparative proteomic analyses on LDs showed that Cox-2 expression was altered in the FFA-induced C2C12cells. More importantly, this alteration was in parallel with FFA-induced insulin resistance (Fig. 5A, lanes 2, 4, and 8). To determine whether Cox-2 is a potential mediator of PA-induced insulin resistance, detailed kinetic experiments were carried out. As shown in Fig. 5B,C, PA-induced Cox-2 expression was dose- and time-dependent, which was reciprocally related to the phosphorylation level of Akt, which represents insulin signaling. A concentration of 200 μM PA was the critical does that induced Cox-2 expression and inhibited insulin-stimulated Akt phosphorylation (Fig. 5B, lane 3). The Cox-2 induction and inhibition of Akt phosphorylation both reached maximum responses at approximately 300 μM PA (Fig. 5B, lane 4). Interestingly, the time course experiment showed that PA-induced Cox-2 expression manifested earlier than the inhibitory effect of PA on Akt phosphorylation (Fig. 5C), suggesting the possibility of a causal relationship.

The results of the dose response and time course experiments indicate that Cox-2 may play a role in PA-induced insulin resistance in C2C12 cells (Fig. 5A).If so, inhibition of Cox-2 activity would reduce PA-induced insulin resistance. Therefore, two Cox-2 inhibitor scelecoxib and indomethacin were chosen to test this possibility. Although both inhibitors could reverse PA-reduced Akt phosphorylation in a dose dependent manner, it is evident that celecoxib had much higher potency than indomethacin (Fig. 5D,E). Both inhibitors could down-regulate the protein expression of Cox-2 at very high concentrations(Fig. 5D, lane 11 and 4E, lane 7).However, their ability to recover PA-reduced Akt phosphorylation was not due to a block of Cox-2 expression (Fig. 5D, lane 8 and 4E, lane 6). Thus, inhibition of Cox-2 activity resulted in recovery of PA-reduced Akt phosphorylation, suggesting that Cox-2 mediates PA-induced insulin resistance.

## Discussion

Since its emergence in 2002, SILAC has become a widely used metabolic labeling strategy in quantitative proteomics. The great advantage of SILAC based techniques over other quantitative mass spectrometry–based methods, such as chemical labeling or label-free techniques, is its quantitative accuracy and reproducibility. These benefits derive from the combination of light and heavy metabolically labeled protein samples at the very beginning of the experimental workflow, thus minimizing possible bias or experimental error.

It is well known that the saturated fatty acid PA induces insulin resistance in skeletal muscle^3^,^4^, while unsaturated fatty acid OA is able to reverse this inhibitory effect^4^. However, the protein(s) that mediate the OA-induced insulin sensitization remain to be identified. We attempted to address this question by assessing the fatty acid-induced alterations in protein expression at the whole proteome scale. Our results, which were based on metabolic labeling by SILAC, together with LC-MS/MS analysis, revealed that the expression of 29 proteins (Table 1) induced by PA treatment could be enhanced or suppressed by PA + OA treatment. Some of the proteins identified were previously reported to be linked with free fatty acid treatment and insulin resistance.

For example, adipose differentiation-related protein (ADRP/PLIN2) was previously identified by 2D gel electrophoresis to be up-regulated by palmitic acid in C2C12 cells. OA has been found to be a more potent inducer of ADRP/PLIN2 expression than PA^30^, which places it in the P4 expression pattern group (Table 1). Fatty acid desaturase 1(Fads1) was reported to be associated with insulin resistance^31^. Hemo oxygenase-1 (Hmox1) plays a pro-inflammatory role in driving insulin resistance in the liver and visceral fat compartments^32^. Sequestosome-1(Sqstm1) was reported to participate in IRS-1 insulin signaling^33^. Pyridoxal-dependent decarboxylase domain-containing protein 1 (PDXDC1), a protein involved in vitamin B metabolism, was observed to be down-regulated in high-fat diet (HFD)-induced insulin resistanceliver^34^, which is consistent with our finding that PDXDC1 was down-regulated upon PA treatment. The dataset of protein expression alteration in the treatment of saturated PA or unsaturated OA may provide much more useful information for dissecting the molecular mechanism that governs insulin signaling regulated by saturated and unsaturated FAs.

Among the proteins that responded to fatty acid treatment, Cox-2 showed the greatest alterations in expression level in response to both fatty acids studied (Fig. 3A and Table 1). This finding is in agreement with the results reported by Dr. Kanzaki’s group from their work with C2C12 cells^18^,^35^. Previously, Hsieh *et al.* demonstrated that celecoxib, a Cox-2 selective inhibitor, reduces fructose-induced insulin resistance in rat whole body and muscle^19^. Using C2C12 myoblasts, we showed that celecoxib was able to reverse PA-induced insulin desensitization, restoring Akt phosphorylation (Fig. 5), and thus demonstrating a mechanism by which celecoxib protects muscle from insulin resistance. However, complicating this interpretation, another study found that inhibiting Cox-2 with NS-398 impaired insulin-stimulated Akt phosphorylation and 2-deoxy-D-[(14)C] glucose uptake in palmitate-exposed skeletal muscle cells^35^. Therefore, the specificity of action of these pharmacologic compounds requires consideration to rectify these results.

Importantly, our data revealed that the PA-induced Cox-2 expression was specifically localized on LDs in C2C12 cells (Figs 4 and 5).This result may have important implications for potential functions of LDs in insulin signaling regulation. We speculate that LDs may link cellular glucose and TAG content and thus represent a nexus for the sensing and control of cellular energy homeostasis.

The main unanswered questions raised by this finding revolve around what relevant arachidonic acid metabolites are produced by PA-induced Cox-2. How do these metabolites involve in the regulation of insulin signaling and by what mechanism do they mediate insulin resistance? Since Cox-2 expression in white adipose tissue was identified to play an important role in protecting mice from high fat diet-induced glucose intolerance^36^, the enzyme may have tissue specific functions with opposite roles in different tissues or cells. Therefore, learning the cellular localization of the enzyme and the type of metabolites it generates in a given set of circumstances are the critical factors for understanding its physiological function.

## Materials and Methods

### Materials

The Colloidal Blue Staining Kit, Hochest 33342 and LipidTOX Red were from Invitrogen. Sodium oleate, sodium palmitate, celecoxib and indomethacin were obtained from Sigma-Aldrich. Western lightning plus-ECL reagent was from PerkinElmer. The synthesized qPCR primers used (Tsingke Technologies Inc., Beijing) are listed in Table S4. Antibodies used in this study are listed in Table S5.

### Cell culture

Mouse C2C12myoblasts (American Type Culture Collections, Manassas, VA) were maintained in DMEM (Macgene Biotech., Beijing) supplemented with 10% FBS (Hyclone), 100 U/mL penicillin and 100 mg/mL streptomycin at 37 °C, 5% CO_2_.

### SILAC labeling

To generate triple labeled SILAC conditions, normal DMEM medium deficient in arginine and lysine (Invitrogen) was supplemented with stable isotope-encoded arginine and lysine (Cambridge isotope laboratories). For “heavy” labeling, L-[^13^C_6_,^15^N_4_]-arginine (Arg10) and L-[^13^C_6_,^15^N_2_]-lysine (Lys8) were used, for “medium” labeling, L-[^13^C_6_]-arginine (Arg6) and L-[^2^H_4_]-lysine (Lys4) were used, and for the “light” conditionL-[^12^C_6_,^14^N_4_]-arginine (Arg0) and L-[^12^C_6_,^14^N_2_]-lysine (Lys0) (Sigma) were used. For SILAC cell culture, C2C12 myoblasts were cultured in medium supplemented with 10% dialyzed fetal bovine serum (Invitrogen), 100 mg/mL streptomycin and 100 U/mL penicillin for at least seven cell population-doubling times to achieve complete labeling (97% labeling efficiency) before fatty acid treatment.

### Fatty acid preparation, treatment and protein extraction

Sodium palmitate (PA) and sodium oleate (OA) were prepared as described previously^4^. Briefly, fatty acids were mixed with ethanol to a final concentration of 100 mM. Then the mixture was sonicated on ice at 200W with 10-sec bursts, 3-sec off pulses until the mixture became a milky homogenous solution. Prepared fatty acid stocks were kept at 4 °C and protected from light. For fatty acid treatment, fatty acid stock solution was added to the cell culture medium containing 10% FBS or 2% filter sterilized free fatty acid free bovine serum albumin (BSA) at 60 °C to a final concentration as stated. For the control, the same amount of ethanol was added to the cell culture medium containing 10% FBS or 2% filter sterilized free fatty acid free BSA as a vehicle. All media were cooled to 37 °C before use. Cells were cultured in the fatty acid-containing media for another 12 hours.

In forward SILAC experiments, light labeled cells were treated with PA, heavy labeled were treated with PA + OA, and medium labeled cells were treated with the vehicle control. In reverse SILAC experiments, light labeled cells were treated with PA + OA, heavy labeled cells were treated with PA, and medium labeled cells were treated with the vehicle control (Fig. 1A).

After treatment, the light, medium and heavy labeled cells were washed with ice-cold PBS twice and scraped into lysis buffer (8 M urea, 100 mM Tris, pH 8.5, supplemented with protease inhibitor cocktail tablet (Roche)) and sonicated. After centrifugation for 30 min at 20,000*g* in a bench-top centrifuge, the supernatants were collected. The protein concentrations in the cell lysates were measured using the Quick Start^TM^ Bradford Protein Assay (Bio-Rad, Hercules, CA).

### SDS-PAGE separation and in-gel digestion

An equi-mass mixture of light, medium and heavy lysates was separated on a 12% SDS-PAGE gel with a 5% stacking gel and was stained with coomassie blue. The gel was cut into 30 slices, and the proteins in the individual gel slices were separately reduced in-gel with dithiothreitol and alkylated with iodoacetamide. The proteins were subsequently digested at 37 °Cwith trypsin (Promega, Madison, WI) overnight. Following digestion, peptides were extracted from gels with 0.1% FA and then with 50% ACN/0.1% FA. The resulting peptide mixtures were dried and stored at −80 °C until further LC-MS/MS analysis.

### LC-MS/MS for protein identification and quantification

LC-MS/MS analysis of SILAC labeling peptide mixtures was performed on an LTQ-Orbitrap XL mass spectrometer with a nanoelectrospray ionization source (Thermo, San Jose, CA) coupled with an Eksigent nano HPLC system. The sample injection, desalting and HPLC separation were conducted automatically on a homemade trap column (150 μm × 50 mm) and an analytical C18 column (150 mm × 75 μM, sunchrom C18 resin, 5 μM, Friedrichsdorf, Germany). The peptide mixture was first loaded onto the trap column with mobile phase A (2% ACN/0.1% formic acid) at a flow-rate of 2.0 μl/min. Then the peptides were separated using a 120 min gradient at a flow rate of 250 nL/min. The 120 min gradient consisted of an 80min gradient from 4% to 30% buffer B (100% ACN/0.1% formic acid), a 20 min gradient from 30 to 45% buffer B, a 5 min gradient from 45 to 80% buffer B, 5 min of 100% buffer B, and finally 10 min of buffer A.

The LTQ-Orbitrap XL mass spectrometer was operated in the positive ion mode, with the high spraying voltage of 2.1 kV. All MS/MS spectra were acquired in a data-dependent scan mode, where one full-MS scan was followed with ten MS/MS scans. The full-scan MS spectra (300~1800 m/z) were acquired with a resolution of 60,000 at m/z 400 after accumulation to a target value of 1,000,000. The ten most abundant ions found in MS^1^ at a threshold above 5,000 counts were selected for fragmentation by collision-induced dissociation at a normalized collision energy of 35%.

### Mass spectrometric data analysis by MaxQuant

The LC-MS/MS data for the identification and quantification of the global proteome were analyzed using MaxQuant^37^ version 1.2.2.5 against the UniportKB mouse database (released on September, 2012) with 75,845 entries to which 175 commonly observed contaminants and all the reverse sequences were added. The maximum number of miss-cleavages by trypsin was set as 2 for peptides. Cysteine carbamidomethylation was set as a fixed modification. N-acetylation (protein) and methionine oxidation were set as variable modifications. The tolerance in mass accuracy for MS and MS/MS were 10 ppm and 0.5 Da, respectively. Only those proteins with at least two distinct peptides detected in the LC-MS/MS analyses were considered reliably identified. The protein expression ratio reported in the present study represented the normalized ratios determined by MaxQuant. The required false positive rate was set to 1% at both the peptide and protein levels, with the minimal required peptide length being set at 6 amino acids. The quantification was based on three independent SILAC experiments and LC-MS/MS analysis, including two forward and one reverse SILAC labelings. The ratio obtained for each individual protein was then normalized against the average ratio for all quantified proteins as described previously^38^. Only those proteins with alterations in expression levels being greater than 2-fold or less than 0.5-fold, and quantified at least twice in the three SILAC labeling experiments were considered as significantly changed.

### Quantitative real-time PCR and Western blotting

Total RNA was extracted with TRIzol reagent and reverse transcribed into cDNA according to the manufacturer’s protocol (Invitrogen, Carlsbad, CA). Quantitative real-time PCR analysis was performed using TheABI StepOne PLUS and SYBR Green detection kit according to the manufacturer’s instructions (Applied Biosystems).

For Western blotting, the indicated proteins were separated by SDS-PAGE and electro-transferred to PVDF membranes, which were blocked with 5% non-fat milk and then probed with primary antibodies. The protein bands were visualized with enhanced chemiluminescence substrate after probing with the indicated secondary antibodies. GADPH was used as loading control.

### Lipid droplet purification, Colloidal blue staining and protein identification with MS

LDs were purified using the method described previously with modifications^39^. Briefly, 10^9^ C2C12 myoblasts with the indicated FFAs treatment were collected after 3 rinses with ice-cold PBS. Then all the cells were transferred to 50 mL buffer C (25 mM tricine pH 7.6, 250 mM sucrose) containing 0.5 mM PMSF. After centrifugation at 1,000*g*, the cell pellets were resuspended in 50 mL buffer C containing 0.5 mM PMSF and were incubated on ice for 20 min. The swelled cells were then homogenized by N_2_ bomb (500 psi for 15 min on ice). The cell lysate was centrifuged at 3,000 *g*, the post-nuclear supernatant (PNS) fraction was collected and loaded into a SW40 tube and was overlain with buffer D (20 mM HEPES, pH 7.4, 100 mM KCl, and 2 mM MgCl_2_). The sample was centrifuged at 250,000 *g* for 1 h at 4 °C. The white band containing LDs at the top of gradient was collected into a 1.5 mL centrifuge tube. The sample was centrifuged at 20,000 *g* for 10 min at 4 °C and then the underlying solution was carefully removed. The droplets were gently resuspended in 200 μL buffer D. This procedure was repeated four times. The lipid extraction and the protein precipitation were carried out by chloroform/acetone (1:1, v/v) treatment followed by centrifuging the sample at 20,000 *g* for 30 min at 4 °C. The protein pellet was then dissolved in 2 × sample buffer (125 mM Tris Base, 20% glycerol, 4% SDS, 4% β-mercaptoethanol and 0.04% bromophenol blue). Following the ultracentrifugation step, the pellet was collected as total membranes (TM) and the infranatant was collected as cytosol (Cyto).

The quality of the LD preparation was evaluated by examining the total protein profile compared with that from the other fractions in silver stained gels. The enrichment or depletion of specific marker proteins was also measured by Western blotting.

For colloidal blue staining, the LDs fractions were separated on a 12% SDS-PAGE with a 5% stacking gel. The gel was then stained with colloidal blue according to the manufacturer’s protocol (Invitrogen). Then the indicated differentially expressed bands between PA and PA + OA treated LDs were cut and subjected to in-gel digestion as described above. The resulting peptide mixtures were separated with C_18_ analytical column and analyzed with LTQ ion trap mass spectrometer. The SEQUEST algorithm was used to search the MS/MS raw spectra against the UniportKB mouse database (2012-09 release) with 175 commonly observed contaminants. The database search was performed using the following parameters for peptide identification: enzyme: trypsin; two missed cleavage sites were allowed, a fixed modification of 57.02 on cysteine, a variable modification of 15.99 on methionine, a mass tolerance of 3.0 Da for peptides (average mass) and 1.0 Da for fragment ions (monoisotopic mass).For protein identification, the search results were filtered with Xcorr versus Charge values of Xcorr ≥ 2.5 for 2 charge peptides, and Xcorr ≥ 3.5 for 3 charge peptides, SP score > 500, RSp < 5, peptide probability ≤0.001, at least two distinct peptides for valid identifications.

### Construction of cox-2 plasmid and immunofluorescence

The mouse cox-2 coding sequence was cloned into pcDNA3.1(+)-myc/hisA with *KpnI* and *XhoI* restriction endonucleases. The forward primer used was: 5′-ATGGTACCGCCACCATGCTCTTCCGAGCTGTGCTGCT-3′; the reverse primer used was: 5′-ATCTCGAGCAGCTCAGTTGAACGCCTTT-3′. The plasmid was sequenced right by TsingkeTechnologies Inc. The plasmid was transfected into cells using electroportion according to the manufacturer’s instructions (Amaxa Nucleofector). Transfected cells were cultured on coverslips in 24-well plates. For immunofluorescence, cells were treated with the fatty acid indicated for 12 hours at 24 hours post transfection. After the fatty acid treatment, cells were rinsed three times with ice-cold PBS and fixed with 4% paraformaldehyde in PBS for 20 min at room temperature. Cells were then rinsed three times with ice-cold PBS, followed by permeabilization in PBS containing 0.1% Triton X-100 for 10 min at room temperature. After rinsing a further three times with PBS, the cells were incubated with 1% BSA for blocking. The cells were rinsed three times with PBS and were then incubated with primary antibody (1:500 dilution, dissolved in 0.3% BSA/PBS) for 1 hour at room temperature. Cells were then washed three times with PBS, 5 min each time. After washing with PBS another three times, the cells were incubated with fluorescein isothiocyanate-conjugated secondary antibody (1:200 dilution, dissolved in 0.3% BSA/PBS) for an additional 1 hour at room temperature and were then washed an additional three times with PBS. For nuclear and LD staining, the cells were incubated with Hochest 33342 and LipidTOX Red, respectively, for an additional 30 min and were then washed three times with PBS, 5 min per wash. The coverslips were finally incubated in antifade solution (Applygen Technologies Inc., Beijing, China) and sealed on a slide with nail polish. Images were captured with an Olympus FV1000 fluorescence confocal microscope (Olympus Corp, Lake Success, NY).

### Statistical analyses

Statistical analyses were performed using GraphPad Prism 6. Results were expressed as mean ± SEM, as indicated in the figure legends. Comparisons between groups were performed using one-way ANOVA.

## Additional Information

**How to cite this article**: Chen, X. *et al.* Comparative Proteomic Study of Fatty Acid-treated Myoblasts Reveals Role of Cox-2 in Palmitate-induced Insulin Resistance. *Sci. Rep.* **6**, 21454; doi: 10.1038/srep21454 (2016).

## Supplementary Material

Supporting information

Supplementary Table S1

Supplementary Table S2

## Figures and Tables

**Table 1:** Representative lists of proteins whose expression of PA treatment could be enhanced or recovered or partially recovered in PA + OA treatment

Pattern	Protein Description	Gene names	Ratio (PA/ctrl)	Ratio(PA + OA/ctrl)
**P4**	Fatty acid desaturase 1	Fads1	2.10 ± 1.44	3.75 ± 2.03
	Adipose differentiation-related protein	Plin2	3.86 ± 0.42	17.53 ± 3.80
	Apob protein	Apob	11.37 ± 9.19	3.56 ± 4.15
	Putative uncharacterized protein Pzp	Pzp	14.31 ± 11.17	8.61 ± 12.92
	Junction plakoglobin	Jup	14.57 ± 13.99	6.96 ± 9.66
**P6**	N-myc downstream regulated gene 1	Ndrg1	2.88 ± 0.23	0.89 ± 0.09
	Sequestosome-1	Sqstm1	7.80 ± 0.95	1.84 ± 0.08
	Caspin	Sdf3	5.23 ± 0.26	1.77 ± 0.34
	Choline kinase alpha	Chk	3.98 ± 0.15	1.09 ± 0.09
	Cystathionine gamma-lyase	Cth	2.03 ± 0.09	1.01 ± 0.22
	Interferon-related developmental regulator 1	Ifrd1	3.60 ± 0.17	1.59 ± 0.20
	Cyclooxygenase-2	Cox2	13.5 ± 2.73	3.71 ± 0.83
	Heme oxygenase 1	Hmox1	8.00 ± 1.30	3.51 ± 0.55
	Spp1 protein	Spp1	6.73 ± 0.70	2.03 ± 0.45
	Putative uncharacterized protein Obsl1	Obsl1	15.97 ± 0.32	1.07 ± 0.03
	Mitogen-regulated protein 1	Mrp1	4.76 ± 0.76	2.44 ± 0.10
	Histone H1.4	H1f4	3.77 ± 2.51	1.33 ± 0.12
	Tissue inhibitor of metalloproteinase 1;	Timp1	3.63 ± 1.78	1.75 ± 0.28
	Alpha-1 type I collagen	Col1a1	6.31 ± 7.11	0.81 ± 0.84
	Cxcl1 protein	Cxcl1	5.43 ± 3.25	2.45 ± 1.31
	Aggrus	Gp38	12.5 ± 4.72	2.10 ± 1.01
	Acyl-CoA thioesterase 2	Acot2	6.23 ± 2.59	2.29 ± 1.08
**P8**	Thymidine kinase	Tk1	0.33 ± 0.08	0.77 ± 0.05
	E-selectin ligand 1	esl-1	0.40 ± 0.21	1.12 ± 0.41
	Reticulocalbin-2	Rcn2	0.41 ± 0.26	0.86 ± 0.09
	Ring finger protein 213	Rnf213	0.32 ± 0.07	0.68 ± 0.11
	Pyridoxal-dependent decarboxylase domain-containing protein 1	Pdxdc1	0.43 ± 0.29	0.85 ± 0.06
	Protein associated with Tlr4	Cnpy4	0.34 ± 0.04	0.67 ± 0.26

**Table 2:** Identification of three protein bands altered in PA-and OA-treatment.

BAND NO.	Protein Name	Gene Name	Sequence Coverage	PEPTIDE NO. (≥5)	MW (KD)	GI NO.
**1**	Cyclooxygenase-2	Cox-2	43.87	29	69.0	31981525
	Heat shock cognate 71 kDa protein	Hspa8	15.94	18	70.9	31981690
	Long-chain-fatty-acid--CoA ligase 3	Acsl3	29.17	17	80.5	209977074
	Cyclooxygenase-1	Cox-1	23.26	14	69.0	6679537
	Ribophorin I	Rpn1	14.14	6	68.5	282398108
	Long-chain-fatty-acid--CoA ligase 4	Acsl4	5.77	5	74.3	75992925
**2**	TCP-1-gamma	Cct3	38.72	36	60.6	6753320
	T-complex protein 1 subunit alpha	Tcp1	27.70	17	60.4	110625624
	Very long-chain specific acyl-CoA dehydrogenase, mitochondrial	Acadvl	17.53	10	70.9	23956084
	Protein-tyrosine phosphatase MEG2	Ptpn9	14.84	10	68.0	61098100
	AMPK subunit alpha-1	Prkaa1	21.65	12	63.9	94681061
	EH domain-containing protein 4	Ehd4	15.71	9	61.5	31981592
	Cct6a protein	Cct6a	9.98	9	58.0	6753324
	Uncharacterized aarF domain-containing protein kinase 2	Adck2	12.97	7	70.2	68649.0
	T-complex protein 1 subunit epsilon	Cct5	10.17	6	59.6	6671702
**3**	Sterol-4-alpha-carboxylate 3-dehydrogenase, decarboxylating	Nsdhl	38.95	13	40.7	31982437
	Annexin A2	Anxa2	24.78	7	38.7	6996913
	Dehydrogenase/reductase SDR family member 1	Dhrs1	10.22	5	34.0	31980844
	UPF0554 protein C2orf43 homolog isoform 1		13.80	5	37.4	269995975
	NADH-cytochrome b5 reductase 3	Cyb5r3	18.27	5	34.1	19745150
	Dehydrodolichyl diphosphate synthase	Dhdds	3.90	5	38.5	27754093
