# Supplementary material for: Comparative Proteomic Study of Fatty Acid-treated Myoblasts Reveals Role of Cox-2 in Palmitate-induced Insulin Resistance
Source: Sci Rep. 2016 Feb 22;6:21454. doi: 10.1038/srep21454 (PMC4761885; doi:10.1038/srep21454)
Supplement: Supporting information [file srep21454-s1.pdf]

Comparative Proteomic Study of Fatty Acid-treated Myoblasts Reveals Role of Cox-2 in Palmitate-induced Insulin Resistance

Supporting Information

Xiulan Chen[1#], Shimeng Xu[2,3#], Shasha Wei[1,3], Yaqin Deng[2,3], Yiran Li[2,4], Fuquan Yang[1*], and Pingsheng Liu[2*]

[1]Laboratory of Protein and Peptide Pharmaceuticals & Laboratory of Proteomics, Institute of Biophysics, Chinese Academy of Sciences, Beijing 100101, China;

[2]National Laboratory of Biomacromolecules, Institute of Biophysics, Chinese Academy of Sciences, Beijing, 100101, China

[3]University of Chinese Academy of Sciences, Beijing, 100101, China

[4]Department of Biological Science and Biotechnology, School of Biological Science and Medical Engineering, Beihang University, Beijing, 100191, China

[#]These authors contributed equally to this work.

*Correspondence to:

Pingsheng Liu, Email: pliu@ibp.ac.cn, Tel.: +86-10-64888517, Fax: +86-10-64888517

Fuquan Yang, Email: fqyang@sun5.ibp.ac.cn, Tel.: +86-10-64888581

TABLE OF CONTENTS

	Pages
All quantified proteins (Table S1)	separate Excel document
Pattern analyis (Table S2)	separate Excel document
Identification of three protein bands altered in PA-and OA-treatment (Table S3)	S3
Primers for qRT-PCR in this study (Table S4)	S5
Information of antibodies used in this study (Table S5)	S6

Table S3 Protein identified in the lipid droplet

Band NO.	Protein name	Accession number	number of peptides	P (probability)	Score	Sequence Coverage(%)	M.W. kDa
1	Prostaglandin G/H synthase 2	Q05769	26	1.00E-30	200.30	43.87	69.0
	Heat shock cognate 71 kDa protein	P63017	14	5.52E-10	140.26	15.94	70.9
	Long-chain-fatty-acid--CoA ligase 3	Q9CZW4	16	3.71E-10	140.23	29.17	80.5
	Prostaglandin G/H synthase 1	P22437	13	6.66E-15	100.30	23.26	69.0
	Dolichyl-diphosphooligosaccharide--protein glycosyltransferas	Q91YQ5	5	1.74E-08	50.20	14.14	68.5
	Heat shock-related 70 kDa protein 2	P17156	3	4.74E-06	30.18	6.00	69.6
	Long-chain-fatty-acid--CoA ligase 5	Q8JZR0	3	2.86E-05	30.17	5.71	76.2
	Long-chain-fatty-acid--CoA ligase 4	Q9QUJ7	3	3.70E-07	30.17	5.77	79.1
	UBX domain-containing protein 4	Q8VCH8	2	2.90E-07	20.21	6.72	56.5
	Sec1 family domain-containing protein 2	Q8BTY8	2	3.80E-05	20.20	6.14	74.8
	Clathrin interactor 1	Q99KN9	2	8.51E-08	20.18	5.23	68.5
	4F2 cell-surface antigen heavy chain	P10852	2	3.82E-04	20.17	4.75	58.3
2	T-complex protein 1 subunit gamma	P80318	30	1.50E-11	268.24	38.72	60.6
	T-complex protein 1 subunit alpha	P11983	16	1.17E-08	150.25	27.70	60.4
	5'-AMP-activated protein kinase catalytic subunit alpha-1	Q5EG47	11	2.47E-11	100.25	21.65	63.9
	Very long-chain specific acyl-CoA dehydrogenase, mitochondri	P50544	9	5.78E-08	90.23	17.53	70.9
	EH domain-containing protein 4	Q9EQP2	9	3.53E-08	90.19	15.71	61.5
	Tyrosine-protein phosphatase non-receptor type 9	O35239	7	2.21E-08	70.20	14.84	68.0
	CDP-diacylglycerol--glycerol-3-phosphate 3-phosphatidyltransf	Q8BHF7	6	2.89E-09	60.27	11.75	62.5
	Uncharacterized aarF domain-containing protein kinase 2	Q6NSR3	5	9.34E-09	60.24	12.97	68.7
	T-complex protein 1 subunit zeta	P80317	6	2.14E-09	50.27	9.98	58.0
	Serine/threonine-protein phosphatase 2A 65 kDa regulatory sub	Q76MZ3	5	5.04E-08	50.18	11.71	65.3
	T-complex protein 1 subunit epsilon	P80316	4	6.50E-09	40.25	10.17	59.6
	Aladin	P58742	4	4.41E-07	40.20	10.81	59.4
	Coatomer subunit delta	Q5XJY5	3	1.03E-07	30.25	7.05	57.2
	Ras GTPase-activating protein-binding protein 1	P97855	3	2.84E-07	30.23	9.25	51.8
	Prolyl 4-hydroxylase subunit alpha-1	Q60715	3	9.05E-10	30.22	7.12	60.9
	T-complex protein 1 subunit zeta-2	Q61390	3	6.41E-11	26.25	5.84	58.2

Band NO	Protein name	Accession nun	number of peptides	P (probability)	Score	Sequence Coverage(%)	M.W. kDa
2	Meckel syndrome type 1 protein homolog	Q5SW45	2	3.61E-08	20.22	5.70	64.4
	Prenylcysteine oxidase-like	Q8C7K6	2	8.16E-07	20.22	4.44	54.9
	FAS-associated factor 2	Q3TDN2	2	1.42E-09	20.21	6.07	52.5
	Heterogeneous nuclear ribonucleoprotein K	P61979	2	8.82E-05	20.20	7.78	51.0
	EH domain-containing protein 1	Q9WVK4	2	6.37E-06	20.19	4.31	60.6
	Patatin-like phospholipase domain-containing protein 2	Q8BJ56	2	2.11E-05	20.18	4.94	53.7
	Acid sphingomyelinase-like phosphodiesterase 3b	P58242	2	9.60E-05	20.17	6.36	51.6
	Lysophosphatidylcholine acyltransferase 1	Q3TFD2	2	1.26E-07	20.16	3.75	59.7
	Squalene monooxygenase	P52019	2	2.05E-06	20.16	4.20	63.8
	Nicalin	Q8VCM8	2	1.22E-05	20.16	3.73	62.9
3	Sterol-4-alpha-carboxylate 3-dehydrogenase, decarboxylating	Q9R1J0	15	4.22E-14	130.25	38.95	40.7
	Annexin A2	P07356	7	6.60E-08	70.21	24.78	38.7
	UPF0554 protein C2orf43 homolog	Q8BVA5	4	4.72E-09	40.21	13.80	37.4
	Choline-phosphate cytidylyltransferase A	P49586	4	1.31E-07	40.20	14.17	41.7
	Phosphatidylserine decarboxylase proenzyme	Q8BSF4	3	4.88E-08	30.23	11.08	45.9
	60S acidic ribosomal protein P0	P14869	3	4.58E-06	30.19	10.73	34.2
	Dehydrogenase/reductase SDR family member 1	Q99L04	2	1.76E-10	20.27	10.22	34.0
	Vesicular integral-membrane protein VIP36	Q9DBH5	2	1.53E-07	20.22	6.70	40.4
	Lactadherin	P21956	2	7.60E-08	20.21	6.91	51.2
	Inactive hydroxysteroid dehydrogenase-like protein 1	Q8BTX9	2	1.01E-08	20.19	8.18	36.9
	Dehydrodolichyl diphosphate synthase	Q99KU1	2	5.21E-06	20.18	3.90	38.5
	3 beta-hydroxysteroid dehydrogenase type 7	Q9EQC1	2	3.97E-05	20.18	5.69	41.1

Table S4. Primers for qRT-PCR in this study

qRT-PCR primers		
Gene	Forward	Reverse
β-Actin	TCCTGTGGCATCCATGAAACT	TGGTACCACCAGACAGCACTGT
Acot2	TCAACGACGCAAAATGGTGG	AGCGGCGGAGGTACAAAC
Apob	GAAGTGTCCAGCCCCATCAC	TGCTGCTCCTTGGCAGTATT
Chkα	TCAGTGTCATCAGGGGTGGT	CTGAGCTTGTTCGGATCCCTC
Cox-1	CCGAGAGATGCGCCTACAG	GCCATCTCCTTCTCTCCTGTG
Cox-2	TGGGTGTGAAGGGAAATAAGGA	ATTTGAGCCTTGGGGGTCAG
Colla1	CCCAATGGTGAGACGTGGAA	TTGGGTCCCTCGACTCCTAC
Fads1	CAGTAGAGCGAATGGGCCTC	CAACCTGCCTGAGCCTGAAC
Glg1	AGATGCTGGATTACCGACGC	CAGTGTCTGAAGCGCCTGTT
Gp38	AATGCAGGGGATGAAACGCA	CTTTAGGGCGAGAACCTTCCA
Hmox1	CCAGAGAAGGCTTTAAGCTGGT	GTGGGGCATAGACTGGGTTC
Obsl1	CAGAATGGTTCAAGCCGCAC	GCTCTGTCTCTCGAACGTGG
Pedf	CTTCAAGGGGCAGTGGGTAA	CAGAGTCCAAGCCGTATCGT
Plin2	CTCTCCTGTTAGGCGTCTCTT	CCTTCTCGGCCATCTCACAC
Sqstm1	AGATGCCAGAATCGGAAGGG	GAGAGGGACTCAATCAGCCG

Table S5. Information of antibodies used in this study

Protein	Host	Source/Manufacturer	Category NO./Remarks
Atp5b	Mouse	Abcam	ab14730
Prohibitin-2	Rabbit	Upstate	07-234
Caspase-3	Rabbit	Cell signaling technology	9665S
Cox-2	Rabbit	Lemin ZHENG's Lab	
Sqstm1	Rabbit	Cell signaling technology	5114S
Cpt-1	Mouse	Abcam	ab128568
Hmox1	Rabbit	Abcam	ab85309
GAPDH	Mouse	Millipore	MAB374
Plin2	Rabbit	Abcam	ab108323
Plin3	Goat	Abcam	ab118605
GRP78	Mouse	BD Transduction	610979
p62	Rabbit	Dr.Dorothy Mundy	
Annexin A2	Mouse	BD Transduction	610069
Tim23	Mouse	BD Transduction	611223
Lamp1	Rabbit	Cell signaling technology	3243s
p-Akt	Mouse	Cell signaling technology	4051s
Myc	Mouse	Jie TANG's Lab	
HRP-conjugated anti-mouse IgG	Goat	ZSGB-BIO	ZB-2305
HRP-conjugated anti-rabbit IgG	Goat	ZSGB-BIO	ZB-2301
HRP-conjugated anti-goat IgG	Rabbit	ZSGB-BIO	ZB-2306
FITC-conjugated anti-mouse IgG	Goat	ZSGB-BIO	ZF-0312
